# Risk prediction models for diabetic retinopathy: a systematic review

**DOI:** 10.3389/fendo.2025.1556049

**Published:** 2025-07-11

**Authors:** Hui Huang, Yingmin Wu, Hejiang Ye, Jiaoyang Li, Ling Chen, Xuan Huang

**Affiliations:** ^1^ School of Management, Chengdu University of Traditional Chinese Medicine, Wenjiang, Chengdu, Sichuan, China; ^2^ Department of Ophthalmology, Hospital of Chengdu University of Traditional Chinese Medicine, Jinniu, Chengdu, Sichuan, China

**Keywords:** diabetes, diabetic retinopathy (DR), predictive modeling, risk factors, systematic review

## Abstract

**Background:**

Diabetic retinopathy, a prevalent complication of *diabetes mellitus*, is a growing public health concern. The use of robust predictive models can aid healthcare professionals in identifying high-risk patients, enabling them to implement early intervention and treatment strategies.

**Objective:**

To systematically evaluate published prediction models for diabetic retinopathy, select better prediction models for healthcare professionals, and provide a valuable reference for model optimization.

**Methods:**

A comprehensive search was conducted across the PubMed, Web of Science, Embase, and the Cochrane Library databases for relevant literature on predictive models for diabetic retinopathy. The search period was set from the time of library construction to November 14, 2023. Furthermore, risk of bias and applicability assessment of the included study models were performed using the PROBAST risk assessment tool.

**Results:**

A total of 2030 studies were retrieved, including 15 studies. The range of the working characteristic curve of the subjects for the 15 models varied from 0.700 to 0.960. All 15 included studies were recognized as high risk of bias. However, five studies had better applicability. The 15 models had Common risk factors for the 15 models included diabetes duration, age, glycosylated hemoglobin, serum creatinine and urinary albumin creatinine ratio.

**Conclusions:**

While the performance of the 15 models had certain predictive performance, the high risk of bias is a concern. Hopefully, future studies will ensure transparency and science in the model-building process by conducting large-sample integrated machine learning, reinforcing multicenter external validation.

This study was registered with PROSPERO, an international prospective systematic evaluation registry platform, and the title was approved with registration number CRD42023483749.

**Systematic review registration:**

https://www.crd.york.ac.uk/PROSPERO/, identifier CRD42024559392.

## 1 Introduction

Diabetic retinopathy (DR) is a prevalent microvascular complication of both type 1 and type 2 diabetes and one of the leading causes of blindness in working adults (1, 2). Global surveys indicated that the number of individuals diagnosed with diabetic retinopathy was 103 million in 2020, which is expected to reach 161 million by 2045 (3). In China, there are about 140 million individuals diagnosed with diabetes (4), among which the prevalence of diabetic retinopathy in patients with type 2 *diabetes mellitus* is 25% (5). Due to the rising incidence of diabetes and the expanding population of individuals with diabetic retinopathy (2), this condition has emerged as a significant public health concern. During the initial stages, diabetic retinopathy does not exhibit any symptoms; however, as the condition advances, it can result in permanent vision loss and eventually complete blindness (6). Early intervention can successfully delay or alter the progression of diabetic retinopathy (7), making it a particularly significant condition.

Using quantitative research methods, predictive disease modeling can help healthcare professionals assess the patient’s condition and take appropriate interventions and treatments to minimize the harm to the patient suffering from the disease. Diabetic retinopathy prediction models can help clinicians in early screening, diagnosis and treatment planning and limited screening of high-risk patients in resource-constrained settings to mitigate disease progression and protect vision (8). The diabetic retinopathy prediction model also helps clinicians to estimate the risk of diabetic retinopathy in diabetic patients, and to personalize the screening and follow-up of patients (9).There are many studies on clinical prediction models for diabetic retinopathy, but diabetic retinopathy risk prediction models incorporate different risk factors with different predictive performances, and it is not clear whether the models can be generalized. Therefore, this review aims to analyze and evaluate diabetic retinopathy prediction models systematically, and the results of the study provide valuable references for future specification of prediction models.

## 2 Methods

### 2.1 Literature inclusion and exclusion criteria

Literature inclusion criteria were as follows: (1) Study population: studies in which patients with type 2 *diabetes mellitus* were diagnosed; (2) Study content: studies in which a predictive model for type 2 diabetic retinopathy was used (except for models of diabetic retinopathy progression, recurrence, and prognosis) and the process of model establishment, validation, and evaluation was described; (3) Study type: cross-sectional studies, case-control studies, cohort studies; (4) Outcome indicators: studies in which the occurrence of type 2 diabetic retinopathy was used as an outcome indicator (10).

Literature exclusion criteria were as follows: (1) studies that only discussed the risk factors of type 2 diabetic retinopathy without constructing models; (2) informal literature such as conference abstracts, reviews, and gray literature; (3) studies based on systematic evaluations to build a model; (4) cellular level studies; (5) duplicated literature and studies that could not be accessed in the original text; (6) articles are in languages other than English for research.

### 2.2 Literature search strategy

A comprehensive literature search was performed using PubMed, Web of Science, Embase, and the Cochrane Library on predictive models for diabetic retinopathy. The search period was set from the time of library construction to November 14, 2023, with English language search and manual searches also performed. The search terms used were a combination of subject terms and free terms, specifically “Diabetic Retinopathy/Diabetic Retinopathies/Retinopathies, Diabetic/Retinopathy, Diabetic/*Diabetes Mellitus* Retinopathy/Predictive Models/Risk Assessment/Risk Prediction/Risk Score” as the English search term. PubMed served as an exemplar for conducting a detailed search.

#1 Search: “Diabetic Retinopathy”[Mesh] Sort By: Most Recent

#2 Search: ((Diabetic Retinopathies[Title/Abstract]) OR (Retinopathies, Diabetic[Title/Abstract])) OR (Retinopathy, Diabetic[Title/Abstract])

#3 #1 OR #2

#4 Search: (((Predictive Models[Title/Abstract]) OR (Risk Assessment[Title/Abstract])) OR (Risk Prediction[Title/Abstract])) OR (Risk Score[Title/Abstract])

#5 #3 AND #4

P (Population): Patients with type 2 diabetes.

I (Intervention model): Prediction model of retinopathy in type 2 diabetes that were developed and published (predictors ≥ 2).

C (Comparator): No competing model.

O(Outcome): The outcome focused on the occurrence of diabetic retinopathy.

T (Timing): The outcome was predicted after evaluating the personal basic information and laboratory indicators of patients with type 2 diabetes.

S (Setting): The role of the risk prediction model is to predict the probability of developing diabetic retinopathy based on the individual circumstances of patients with type 2 diabetes to prevent adverse events.

### 2.3 Literature screening and information extraction

This review used EndNote 21 literature manager to remove duplicates. Two researchers independently assessed the title and abstract of the literature based on inclusion and exclusion criteria. The full text was examined meticulously to find relevant inclusion of literature in this study, and data was retrieved and verified through cross-checking. In the event of any disagreements, they were resolved through discussion or negotiation with the involvement of a neutral third party. The process of extracting data for systematic reviews of prediction modeling studies, as outlined in the CHARMS checklist (11), involves gathering information on the fundamental characteristics of the literature being reviewed, such as the first author, publication year, country, study type, case collection time, data source, study model type, and sample size. Additionally, details about the predictive model are collected, including how missing values are handled, feature extraction methods, model development techniques, calibration methods, model validation approaches, model performance, and predictors.

### 2.4 Assessment of risk of bias and applicability of prediction models for included studies

Two researchers independently assessed the risk of bias and applicability of the included literature based on the prediction model research risk of bias assessment tool PROBAST (prediction model risk of bias assessment tool) (12, 13). If there is a dispute, it is resolved through discussion or negotiation with a third party. The tool is suitable for researching various diagnostic or prognostic models. PROBAST contains four domains: study population, predictor variables, outcomes, and analysis, with 20 entries in the four domains. Each domain is answered with “low,” “high,” or “unclear.” The four domains of study object, predictor variable, outcome, and analysis were used to assess the risk of bias in the prediction model. The three domains of the study population, predictor variables, and outcomes were used for the assessment of the applicability of the predictive model. Based on the results of each field, the overall risk of bias and applicability of the prediction model were determined, which were reported using the terms “low risk of bias or high applicability,” “high risk of bias or low applicability,” and “unclear risk of bias or unclear applicability.”

### 2.5 Data synthesis and statistical analysis

Meta-analysis of the AUC values of the model was performed using MedCalc software. Heterogeneity was tested using the I² index. I² value of ≤25% indicated low heterogeneity, 25%<I²≤50% indicated moderate heterogeneity, and I²>50% indicated high heterogeneity. Heterogeneity was analyzed according to the results using either a fixed-effects model or a random-effects model, and publication bias was identified using the Egger test, with p > 0.05 indicating a low likelihood of publication bias.

## 3 Results

### 3.1 Literature screening process and results

**Figure 1:**
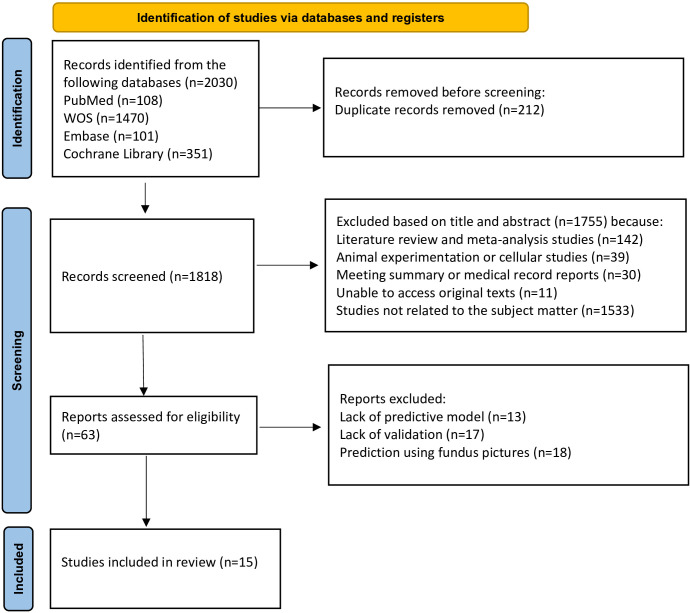
Literature screening process.

A total of 2030 articles were obtained from the literature search using the specified search terms, including 108 articles from PubMed, 101 articles from Embase, 1470 articles from Web of Science, and 351 articles from the Cochrane Library. After removing 212 duplicate articles, the titles and abstracts of 1818 articles were reviewed. Following the screening procedure, 63 articles were selected for additional assessment, and 15 documents (11–25) were included. **Figure 1** depicts detailed results.

### 3.2 Basic characteristics of the included literature

Fifteen studies (14–28) were included, published between 2013–2023, with thirteen studies (14–26) published in the last three years. There were twelve (14–20, 22, 24–27) studies in China, one (21) in the UK and India, and two (23, 28) in South Korea. Of the included studies, eleven (15–17, 19–24, 26, 27) were retrospective cohort studies, and four (14, 18, 25, 28) were cross-sectional studies. Six studies (16, 21, 23–25, 27) were multicenter studies, and nine (14, 15, 17–20, 22, 26, 28) were single-center studies. **Table 1** depicts the detailed results of the analysis.

**Table 1:** Basic characteristics of included studies.

Author year	Countries	Research design	Collection time	Data sources	Type of research models	Sample size
Zong 2023 (14)	China	Cross-sectional study	2015-2016	Liaoning Medical University First Affiliated Hospital	A	1032
Zhang 2023 (15)	China	Retrospective cohort study	2020-2022	the Second Affiliated Hospital of Kunming Medical University.	B	1257
Yang 2023 (16)	China	Retrospective cohort study	2010-2022	one hospital in Chongqing and four hospitals in Chengdu	B	4159
Wang 2023 (17)	China	Retrospective cohort study	2011-2018	the National Health and Nutrition Examination Survey database	B	931
Gong 2023 (18)	China	Cross-sectional study	2019	the Guangdong Shaoguan Diabetes Cohort Study	B	2294
Zhao 2022 (19)	China	Retrospective cohort study	2010-2018	the Dalian Medical University Affiliated	A	7943
Yang 2022 (20)	China	Retrospective cohort study	2016-2021	the Second Hospital of Shijiazhuang	B	5900
Nugawela 2022 (21)	Britain, India	Retrospective cohort study	2007-2017	three Clinical Commissioning Groups Queen Mary University London Secure Anonymised Information Linkage Dr Mohan’s Diabe- tes Specialities Centre Madras Diabetes Research Foundation	B	160515
Li 2022 (22)	China	Retrospective cohort study	2010-2019	the First Affiliated Hospital of Xinjiang Medical University	B	13980
Jo 2022 (23)	South Korea	Retrospective cohort study	2009-2020	Six hospitals in Korea	B	9102
Su 2021 (24)	China	Retrospective cohort study	2015-2020	Datadryad the Aviation General Hospital	A	610
Li 2021 (25)	China	Cross-sectional study	2019	the Chinese PLA General Hospital, the Strategic Support Force Medical Centre	B	906
Li 2021 (26)	China	Retrospective cohort study	2013-2017	the Chinese PLA general hospital	A	32452
Mo 2020 (27)	China	Retrospective cohort study	2015-2018	Six communities in Shanghai	B	4170
Oh 2013 (28)	South Korea	Cross-sectional study	2010-2011	the Korean National Health and Nutrition Examination Survey	B	1052

A: Development + Validation + Comparison.

B: Development + Validation.

### 3.3 Basics of the model

For variable selection among the included studies, four studies (18, 19, 23, 24) screened variables using only a one-way analysis of variance, followed by seven studies (14, 17, 20, 22, 25, 27, 28) that screened predictors directly using LASSO. For model construction, seven studies (14, 19, 23, 24, 26, 28) used multiple modeling methods and determined the final model by comparing the AUC. Regarding missing data, seven studies (15, 18–20, 25, 27, 28) did not report how they treated missing values. Only three studies (14, 16, 22) dealt with missing values using multiple interpolations, missing-then-excluded (17), coding as a separate category (21), regression with supervised machine learning (23), K-nearest-neighbor interpolation (26), and maximum likelihood estimation (24) for one study each. Among the models included in the 15 studies, the main predictors of diabetic retinopathy prediction models were diabetes duration, glycosylated hemoglobin, age, serum creatinine and urinary albumin creatinine ratio. The AUC values of the models ranged from 0.700 to 0.960, indicating that the models had some predictive performance. **Table 2** depicts the detailed results.

**Table 2:** Overview of information on the predictive models included in the study.

Author Year	Missing data handling	Variable selection	Model development method	Calibration method	Validation	Model performance		Predictors
						AUC	95%CI	
Zong 2023 (14)	Multiple imputations	LASSO	Logistic regression XGBoost	Brier Calibration Chart	Internal validation	0.820	0.75–0.82	3-hydroxy-octadecylcarnitine Phenylalanine Octacarbonylcarnitine Threonine Tyrosine
Zhang 2023 (15)	—	Single factor analysis	Logistic regression logistic regression with backward stepwise selection LASSO	—	Internal validation	0.728	0.694—0.762	Duration of diabetes Age at onset Treatment method Total cholesterol Urinary albumin to creatinine ratio Urine sugar
Yang 2023 (16)	Multiple imputations	Single factor analysis LASSO	Logistic regression	Calibration Chart DCA CIC	Internal validation External validation	0.722	0.696–0.748	Duration of diabetes History of hypertension Cardiovascular disease
Wang 2023 (17)	Excluded if missing	LASSO	Logistic regression	Hosmer-Lemeshow DCA Calibration Chart	Internal validation	0.709	0.659—0.759	Gender Taking insulin Duration of diabetes Urinary albumin creatinine ratio Serum phosphorus
Gong 2023 (18)	—	Single factor analysis	Logistic regression	Hosmer-Lemeshow Calibration Chart	Internal validation	0.719	—	Age BMI SBP Duration of diabetes HbA1C
Zhao 2022 (19)	—	Single factor analysis	XGBoost Random Forest Logistic regression Support Vector Machines KNN	Harrell	Internal validation	0.913	0.901–0.925	HbA1c Duration of diabetes Follow-up time Postprandial blood glucose Age
Yang 2022 (20)	—	LASSO Random forest	Logistic regression	Harrell DCA CIC Calibration Chart	Internal validation	0.820	0.802—0.838	Duration of diabetes Diabetic neuropathy Diabetic kidney disease Diabetic foot, hyperlipidemia Hypoglycemic drugs Glycated albumin Lactate dehydrogenase
Nugawela2022 (21)	Coding as a separate category	Single factor analysis Backward elimination procedure	Cox regression	Calibration Chart	Internal validation External validation	0.832	0.822—0.842	Age Gender Duration of diabetes HbA1C Antidiabetic medication history
Li 2022 (22)	Multiple imputations	LASSO	Logistic regression	Harrell Akaik Hosmer-lemeshow DCA Calibration Chart	Internal validation	0.882	0.875–0.888	Peripheral neuropathy Age Neutrophilic granulocyte High-density lipoprotein HbA1C Duration of diabetes Glycosylated serum protein
Jo 2022 (23)	Regression for supervised machine learning	Single factor analysis	Decision trees Logistic regression Support vector machine Naïve Bayes Ensemble decision trees	—	Internal validation	0.960	—	Baseline vision Duration of diabetes treatment Serum level of glycated hemoglobin Creatinine Estimated glomerular filtration rate Blood pressure
Su 2021 (24)	Maximum likelihood estimation	Single factor analysis	Logistic regression BP-ANN	—	External validation	0.880	0.780–0.910	Age Sex Albumin Creatinine Duration of diabetes
Li 2021 (25)	—	LASSO	Logistic regression	Hosmer-Lemeshow	Internal validation External validation	0.820	—	Duration of diabetes Diabetic nephropathy Serum creatinine level Annual DR screening Hyperlipidemia
Li 2021 (26)	KNN interpolation	Recursive Feature Elimination	XGBoost Logistic regression Random Forest Support Vector Machines	—	Internal validation	0.900	—	HbA1c Nephropathy Serum creatinine Insulin treatment Diabetic lower extremity arterial disease
Mo 2020 (27)	—	LASSO	Logistic regression	DCA Hosmer-Lemeshow Calibration Chart	External validation	0.700	—	Age Duration of diabetes Postprandial blood glucose HbA1c Uric creatinine Urinary microalbumin SBP
Oh 2013 (28)	—	—	Ordinary logistic regression Logistic regression with backward stepwise selection Ridge Elastic net LASSO	—	Internal validation External validation	0.810	0.740—0.860	Fasting plasma glucose Triglyceride BMI Insulin therapy

### 3.4 Validation of the model

All fifteen studies validated the model, in which nine studies (14, 15, 17–20, 22, 23, 26) used internal validation, four studies (16, 21, 25, 28) used a combination of internal and external validation and two studies (24, 27) used external validation. The model was mainly calibrated using Hosmer–Lemeshow, calibration graphs, and DCA to calibrate the model, and five studies (16, 23, 24, 26, 28) did not calibrate the model, and the detailed results are shown in **Table 2**.

### 3.5 Risk of bias and applicability results

#### 3.5.1 Risk of bias evaluation

Based on the PROBAST evaluation criteria, all 15 included literature had a high overall risk of bias. The main reasons are reflected in the research object field, outcome field, and analysis field. Subject area: A total of eleven studies (15–17, 19–24, 26, 27) were identified to have a high risk of bias. This was mostly because the retrospective studies used data sources not initially created for modeling and validation purposes. The remaining four studies (14, 18, 25, 28) exhibited a low risk of bias.

Predictor variable domain: Two studies (27, 28) were identified to have a low risk of bias, and thirteen studies (14–26) were identified to have an unclear risk of bias. The main reason was that these studies did not explicitly answer the question “whether the assessment of predictors was made without knowledge of clinical outcome data” and did not report this information.

Outcome areas: Two studies (21, 23) were identified to have a high risk of bias. Nugawela (21) and Jo (23) conducted multi-center retrospective studies with unclear descriptions of clinical outcome assessment and a significant potential for bias. Thirteen studies (14–20, 22, 24–28) were found to have an uncertain level of bias, primarily due to insufficient information regarding the method of predictor assessment and the time of predictor measurement.

Analytic domain: All fifteen studies identified a high risk of bias in the analytic domain. For continuous variable treatment, six studies (14–16, 18, 21, 22) transformed some continuous variables into categorical variables, which may lead to a decrease in the predictive power of the model. Regarding missing data, seven studies (15, 18–20, 25, 27, 28) did not report information on how missing data were handled. Regarding selecting predictive factors, four studies (18, 19, 23, 24) used one-way analysis and did not use appropriate methods to select predictors. Regarding model validation, nine studies (14, 15, 17–20, 22, 23, 26) partitioned a certain amount of data for internal validation and did not conduct external validation to determine the general applicability of the model. **Table 3** presents a detailed data for the analysis.

**Table 3:** Risk of bias and applicability assessment results.

Author year	Risk of bias evaluation				Results of risk assessment of bias	Applicability evaluation			Results of applicability evaluation	Overall evaluation
	Participants	Predictors	Outcomes	Analysis	Results	Participants	Predictors	Outcomes	Results	
Zong 2023 (14)	L	—	—	H	H	L	L	L	L	H
Zhang 2023 (15)	H	—	—	H	H	L	L	L	L	H
Yang 2023 (16)	H	—	—	H	H	L	—	L	—	H
Wang 2023 (17)	H	—	—	H	H	H	—	—	H	H
Gong 2023 (18)	L	—	—	H	H	L	—	L	—	H
Zhao 2022 (19)	H	—	—	H	H	L	L	L	L	H
Yang 2022 (20)	H	—	—	H	H	L	—	L	—	H
Nugawela 2022 (21)	H	—	H	H	H	L	—	H	H	H
Li 2022 (22)	H	—	—	H	H	H	—	—	H	H
Jo 2022 (23)	H	—	H	H	H	L	—	L	—	H
Su 2021 (24)	H	—	—	H	H	L	L	L	L	H
Li 2021 (25)	L	—	—	H	H	L	L	L	L	H
Li 2021 (26)	H	—	—	H	H	L	—	L	—	H
Mo 2020 (27)	H	L	—	H	H	L	—	L	—	H
Oh 2013 (28)	L	L	—	H	H	—	—	—	—	H

H: high risk of bias or low applicability; —: risk of bias unclear or applicability unclear; L: low risk of bias or high applicability.

#### 3.5.2 Evaluation of applicability

Three studies (17, 21, 22) were identified as having a high risk of bias for applicability evaluation, seven studies (16, 18, 20, 23, 26–28) were identified as unclear risk of bias, and five studies (14, 15, 19, 24, 25) were identified as having a low risk of bias.

In the area of the study population, one study (21) lacked information about the study subjects. In the area of predictor variables, ten studies (16–18, 20–23, 26–28) lacked information about the definition of predictors. One study (21) was at high risk of bias for the outcome domain, and Nugawela (21) defined severe nonproliferative diabetic retinopathy as diabetic retinopathy. The definition of diabetic retinopathy in this review referred to the International Clinical Diabetic Retinopathy Severity Scale (10), which included mild nonproliferative diabetic retinopathy, and the different definitions resulted in a high risk of bias. Three studies (17, 22, 28) lacked information about the definition of diabetic retinopathy. **Table 3** depicts a detail.

### 3.6 Meta-analysis results

**Figure 2:**
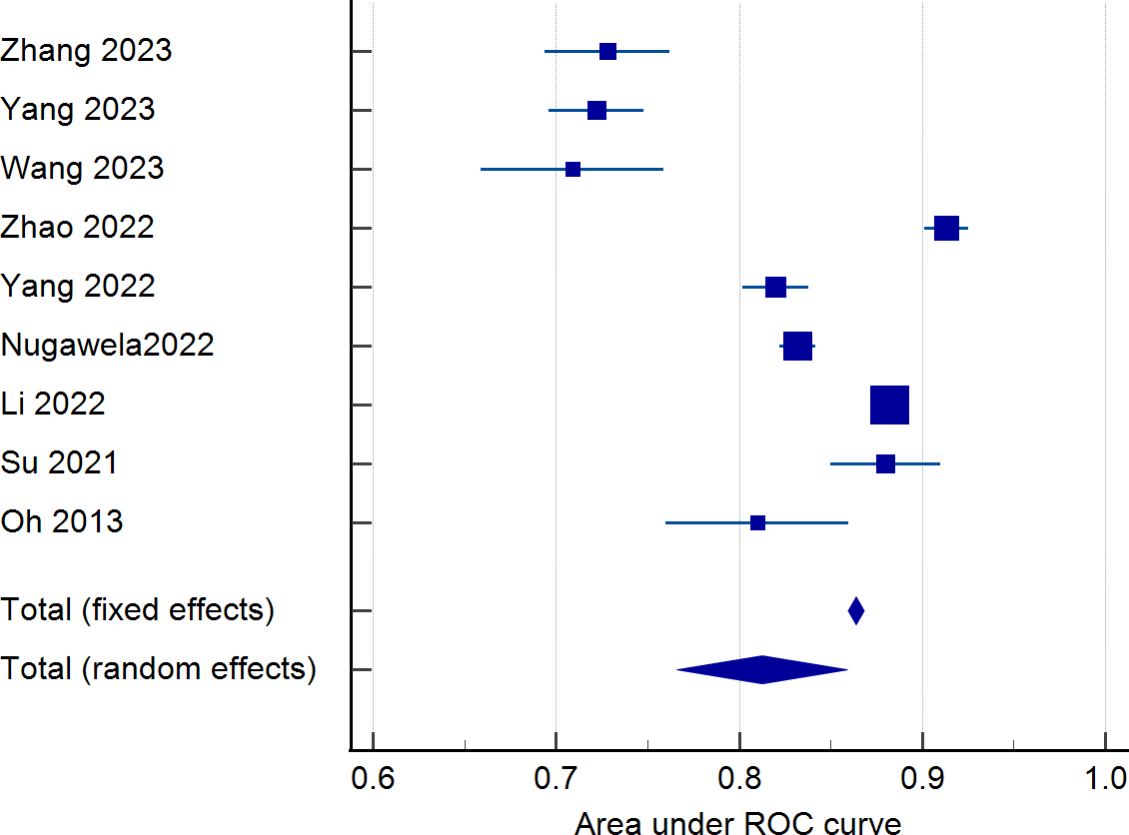
Forest plot of the area under the receiver operating characteristic curve for the risk prediction model.

**Figure 3:**
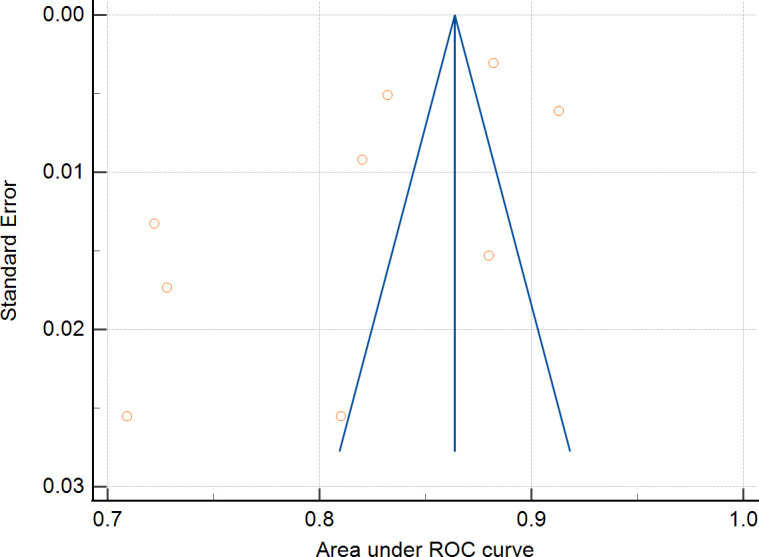
Funnel plot examination.

Nine studies were eligible for pooling due to underreporting of model development details in the included studies. A random effects model was used to calculate the combined AUC, and the analysis resulted in a combined AUC value of 0.812 (95% CI: 0.766-0.859). **Figure 2** depicts detailed results. I^2^ value of 97.89% (p < 0.001) indicated a high degree of consistency between the studies, and an Egger’s value of -6.963 (p=0.06), suggesting that there was no significant publication bias but close to the level of significance, the potential possibility of bias needs to be considered with caution. The funnel plot is shown in **Figure 3**.

## 4 Discussion

A total of fifteen diabetic retinopathy prediction models were included in this study after screening, and the AUC values of the included models ranged from 0.700 to 0.960, which had a certain predictive performance. According to the AUC value, Jo’s model has the strongest predictive performance, which is not only due to the use of ensemble algorithms in the model construction process but also supported by rich multicenter data. However, fifteen studies were evaluated using the risk of bias assessment tool PROBAST, and all were recognized as high risk of bias. Model performance varies widely and model heterogeneity is high. The reasons for this were mainly cases of inappropriate selection of study subjects, inappropriate treatment of continuous variables, inappropriate methods of screening predictor variables, treatment of unreported missing values, and lack of external validation.

Firstly, regarding object of study. The predictive models included in this study were predominantly retrospective studies. Although the sample size is large, predictors in retrospective studies may not be comprehensive, and there are missing data, leading to biased results. Conversely, prospective studies ensure that the measurement of predictors occurs before the outcome and that the predictors are assessed uniformly, enhancing the reliability of the model results. Regarding missing value treatment, only three studies used multiple interpolations to deal with missing data, whereas multiple interpolations can reduce the impact of missing data on statistical analysis and model accuracy (29). When missing data are handled inappropriately, valuable information hidden in the excluded objects may be ignored or lost, leading to model bias; thus, choosing appropriate missing data handling methods in future studies is crucial. Then, regarding predictive variable. Seven studies (14, 17, 20, 22, 25, 27, 28) in this review used LASSO to select predictors. Four studies (18, 19, 23, 24) used only one-way analyses to include statistically significant predictors in the model analyses, which may have omitted significant predictors. Other studies have shown that using LASSO leads to better identification of predictors and improves the predictive performance of the model (30, 31); thus, future studies should use the right approach while selecting predictors. Notably, the included studies used logistic regression and integrated algorithms such as decision trees, random forests to construct predictive models. The predictive performance of constructing models using integrated algorithms was stronger than that of logistic regression, consistent with the results obtained in studies conducted in other areas of medicine (32, 33). However, the issue of overfitting in the process of building models using integrated algorithms still needs to be taken seriously. Internal validation of predictive models aims to test the repeatability of the model and effectively prevent overfitting (34), while external validation is regarded as the “gold standard” for testing the generalizability of the model. While all the research included in this study conducted model validation, many studies focused on internal validation. Only four studies (16, 21, 25, 28) conducted internal and external validation, but the external validation aspect of these studies may be improved. Before clinical implementation, multiple external validations are crucial to assess the model’s stability and overall applicability. This is necessary due to significant variations in baseline characteristics and other factors among different target populations (35). Therefore, future researchers should focus on the external validation of the model to ensure its reliability in practical application.

The final predictors in the fifteen models included in this study ranged from three to seven. Despite differences in the type of study and study area, predictors of each model varied, but there were some commonalities. The most common predictors include duration of diabetes, age, glycosylated hemoglobin, serum creatinine and urinary albumin creatinine ratio. The prevalence of diabetes retinopathy increases with the increase of the course of diabetes. The prevalence of diabetic retinopathy was 9.44% when the duration of diabetes was less than 5 years, and 76.47% when the duration of diabetes was 20–25 years (36). Therefore, patients with a long duration of diabetes are a priority for diabetic retinopathy prevention. The older the patients with diabetes, The risk of diabetic retinopathy is influenced by dual age factors: the patient’s current age and the age of diagnosis of diabetes. Among them, the younger the age of diagnosis, the higher the risk of retinal lesions (37, 38). Therefore, middle-aged people are also the focus of screening for diabetes retinopathy. For this reason, some scholars had established a risk prediction model for diabetes retinopathy in middle-aged patients with type 2 diabetes (17) to predict the risk of diabetes retinopathy in middle-aged people and intervene in advance. Glycated hemoglobin was significantly associated with diabetic retinopathy. According to research, the optimal HbA1c threshold for detecting any diabetic retinopathy was 49 mmol/mol (6.6%) and 52 mmol/mol (6.9%) for moderate or severe retinopathy (39). A meta-study showed that glycosylated hemoglobin has good diagnostic value and validity for diabetic retinopathy because it has the advantages of being more stable than blood glucose and independent of dietary influences (40). Therefore, it is important for people with diabetes to control their blood sugar levels in their daily lives to reduce the likelihood of developing diabetic retinopathy. A multicenter cohort study with 8 years of follow-up found that patients with diabetic retinopathy had higher serum creatinine levels and higher urinary microalbumin/urinary creatinine than patients without diabetic retinopathy (41). Given that most models’ data originate from China, the inferred predictive factors may be more suitable for application in developing countries. In these countries, predictive indicators such as HbA1c, duration of diabetes, and the urinary albumin-to-creatinine ratio (UACR) are not only easily accessible but also relatively low in cost, making them an ideal choice in resource-limited settings. Moreover, these factors have also shown good accuracy in predicting diabetic retinopathy.

Although early intervention is an effective measure to prevent diabetic retinopathy, the performance of the diabetic retinopathy risk prediction model constructed using the above predictors needs to be further validated. Meanwhile, several studies (42, 43) have successfully combined fundus images with machine learning to recognize key features of diabetic retinopathy. Acquiring fundus images provides another important feature for diabetic retinopathy prediction models, which may further enrich the predictive ability and accuracy of the models.

## 5 Limitations

This review has certain limitations. First, most of the research subjects included in this review are from China, and the generalizability of the findings to Western populations may be limited. Second, the current PROBAST assessment has many discomforts for the risk of bias in machine learning-related studies. PROBAST-AI is required (44, 45), but this assessment tool is still under development. Furthermore, considering the heterogeneity of the included studies regarding the type of design, data sources, and modeling methods, no quantitative analysis of the included studies was performed. Finally, this study only included English literature, and research results in other languages were not included.

## 6 Conclusions

To summarize, all 15 prediction models included in this systematic review were thoroughly evaluated and showed robust predictive capabilities. The assessment results of the PROBAST tool indicated that all the predictive models examined in the research were identified as having a significant risk of bias. Future researchers are advised to adhere rigorously to PROBAST guidelines to ensure transparency and scientific accuracy in developing models and to enhance the quality of future studies. In the future, as medical record databases are established and the era of artificial intelligence begins, we will use large-sample integrated machine learning algorithms and deep learning algorithms to train models. This will help us strengthen the external validation of multi-center data and develop prediction models with good predictive performance and applicability.

## Data Availability

The original contributions presented in the study are included in the article/**Supplementary Material**. Further inquiries can be directed to the corresponding author/s.
