# Supplementary figures and images for: Risk prediction models for diabetic retinopathy: a systematic review
Source: Front Endocrinol (Lausanne). 2025 Jul 11;16:1556049. doi: 10.3389/fendo.2025.1556049 (PMC12291684; doi:10.3389/fendo.2025.1556049)

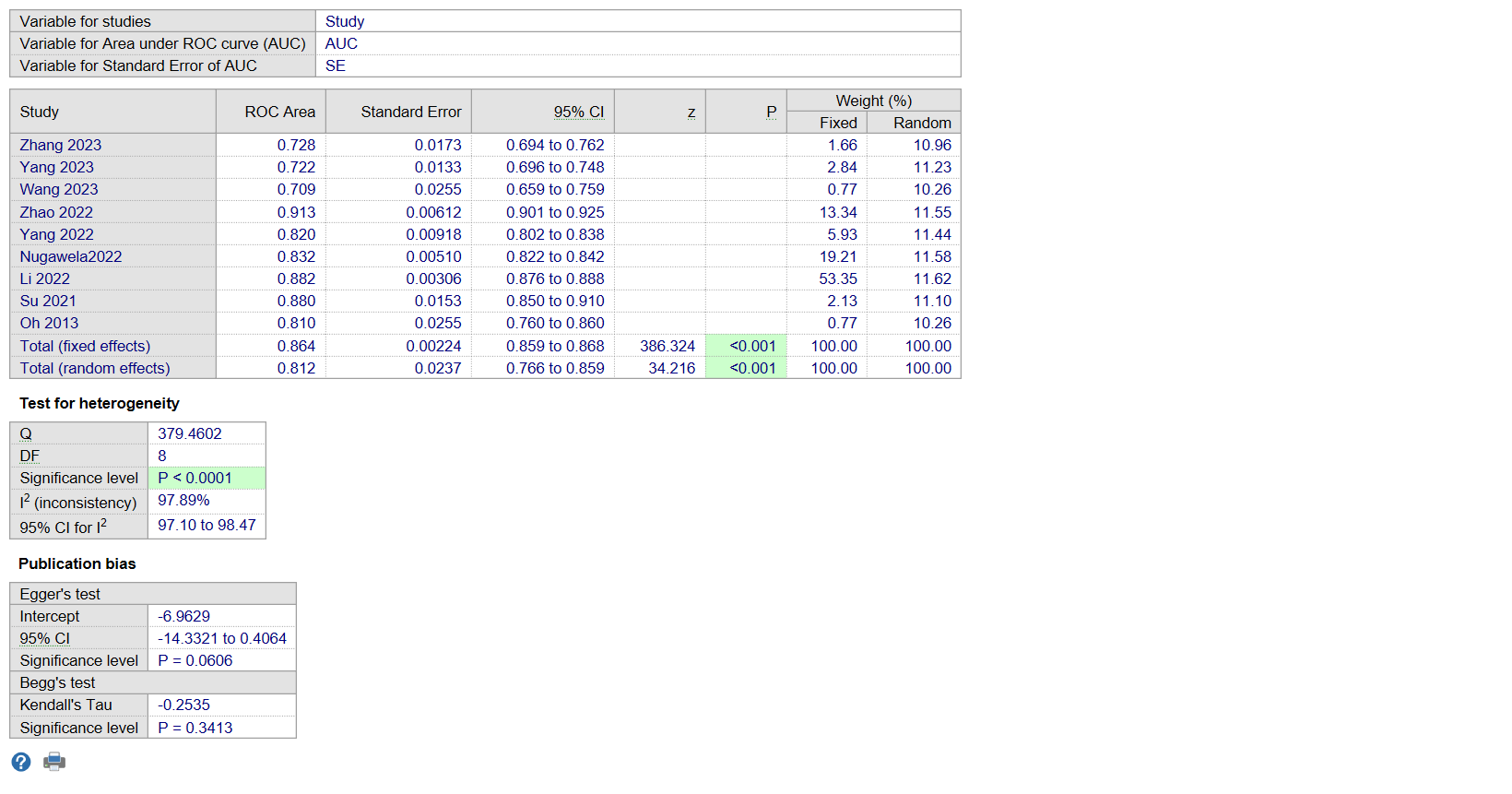

Supplement: Supplementary file 1 [file Image1.jpeg]
